# Effects of different drying processes on the quality changes in Arapgir purple basil (*Ocimum basilicum* L.) leaves and drying‐induced changes in bioactive and volatile compounds and essential oils

**DOI:** 10.1111/1750-3841.17515

**Published:** 2024-11-26

**Authors:** Kadriye Altay, Safiye Nur Dirim, Ali Adnan Hayaloglu

**Affiliations:** ^1^ Ministry of Agriculture and Forestry Olive Research Institute Bornova Izmir; ^2^ Department of Food Engineering, Engineering Faculty Inonu University Malatya; ^3^ Department of Food Engineering, Engineering Faculty Ege University Bornova İzmir

**Keywords:** antioxidant activity, essential oil, purple basil leaves, total phenolic content

## Abstract

The aim of this research is to examine the effects of drying purple basil leaves (*Ocimum basilicum* L.) under different drying conditions (freeze drying, sun‐drying, and convective drying [CD] at 45, 50, and 55°C and microwave drying at 350, 460, and 600 W) on color properties, total phenolic and anthocyanin content, antioxidant activities, and changes in the composition of volatile compounds and essential oils (EOs). Increasing the drying temperature and microwave power led to an improvement in the preservation of the total phenolic content of the samples by an average of 16.28% and 27.98%, respectively. Increasing the drying temperature resulted in lower anthocyanin content and antioxidant activity (AA); in contrast, increasing the microwave power resulted in higher anthocyanin content and AA when drying purple basil. The drying methods significantly changed the composition of volatiles and EOs, and microwave drying resulted in a different profile of volatiles and EO composition. The key volatile compounds in purple basil leaves were linalol (81.19–1176.09 µg/g dw), 1,8‐cineole (45.15–816.16 µg/g dw), and methyl cinnamate (13.20–637.65 µg/g dw). On the other hand, methyl cinnamate (11.68%–57.66%) and linalool (0.02%–20.39%) were the main volatile compounds of EOs in basil leaves. In conclusion, the following drying methods are suitable for the protection of phenolic and anthocyanin compounds and high performance of AA: freeze drying, sun‐drying, and CD at 45°C and microwave drying at 600 W.

## 1 INTRODUCTION

People have been using aromatic and medicinal herbs for therapeutic purposes for a long time. Approximately 250 medicinal plants were already recognized during the Mesopotamian civilization, and this number continues to grow steadily. In recent years, the utilizing medicinal and aromatic plants in the pharmaceutical industry have increased significantly worldwide (Ghorbanpour et al., 2017). It is estimated that 20,000 plant species are used for medicinal purposes worldwide, of which 4000 are widely used. *Ocimum basilicum* L. belongs to the members of the Lamiaceae family of aromatic plants and is among the medicinal plants used for this purpose (Keskin, 2018). Basil is used both medicinally and aromatically due to its properties as an essential oil (EO) and is characterized by its antibacterial, antimutagenic, and antioxidant properties. Its phenolic compounds, flavones, and tannins contribute to its ability to protect against cancer through its anti‐carcinogenic effect. Phenolic substances, which are the most important group of natural antioxidants, protect easily oxidizable substances in food from oxidation (Złotek et al., 2017).

The volatiles in the oil of the basil plant include oxygenated and hydrocarbon monoterpenes, sesquiterpenes, phenylpropanoids, aldehydes, and esters. Linalool, 1,8‐cineole (also known as eucalyptol), methyl chavicol, eugenol, bergamotene, methyl cinnamate, geraniol, geranial, neral, and α‐bergamotene are some common constituents (Shahrajabian et al., 2020; Sonmezdag et al., 2018). Due to various factors, such as climatic and geographical conditions, cultivation techniques, harvest time, age of the plant, and even the extraction process, the amount of each constituent can vary greatly (Babu et al., 2018).

Due to the sensitive structures of medicinal and aromatic plants, the choice of gentle drying methods that are suitable for the product is of particular importance. There are studies in which basil was dried in a tray dryer (Chaves et al., 2022), a microwave dryer (Altay et al., 2019), a vacuum microwave dryer (Łyczko et al., 2020), a freeze dryer (Telfser & Gómez Galindo, 2019), and a vibrofluidized dryer (de Aquino Brito Lima‐Corrêa et al., 2017). Moreover, it has been dried in a natural way under the sun (Sharma et al., 2018) and in the shade (Gurkan & Hayaloglu, 2023). Fresh or dried, it is commonly employed to enrich the aroma and flavor of foods like pizza, salad, meat, pasta, sauce, soup, seafood, confectionery, baked goods, soft drinks, vinegar, ice cream, and cheese (Altay et al., 2019). In our research, purple basil leaves were dried in the sun, in a microwave oven, in a tray dryer, and in a freeze dryer, and the impacts of the various drying conditions and methods on the color and chemical characteristics as well as the volatile and EOs of the samples were investigated.

## 2 MATERIALS AND METHODS

### 2.1 Materials

A geographically registered purple basil (by TURKPATENT in 2017) *O. basilicum* L. was procured from local producers cultivating in the Kozluk Valley of Arapgir district (39°02′N, 38°29′D) of Malatya province, Türkiye. Basils were supplied throughout the harvest season of June to September 2016 and brought to the laboratory immediately after harvesting. Before drying, the stems of the basil were removed, and the leaves were separated and refrigerated at 4°C. Four batches of fresh purple basil leaves (FB) were prepared for the various drying methods. Each drying experiment was carried out three times.

### 2.2 Drying experiments

A series of preliminary trials were carried out by examining the temperatures and microwave powers given in the literature for drying of medicinal and aromatic plants, with a choice between 40 and 60°C for tray drying, 100–900 W for microwave drying, and different drying times for freeze drying (conditions specified on the device) and sun‐drying. Appropriate drying conditions were chosen based upon the moisture, water activity, and color analysis results. The experiment was carried on until the sample achieved a moisture content of 8%. The material was dried repeatedly until the moisture content reached 8%. The FB of moisture content from an initial value of 86.61% (wb) decreased to a final value of 3.09–8.01% (wb), based on the drying method. The data and diagrams on the drying characteristics of FB were mentioned in our previous study (Altay et al., 2019).

A laboratory‐scale freeze dryer (Armfield, FT 33 Vacuum Freeze Drier) was used for the freeze‐drying experiments. The FB, approximately 50 g, each were freeze‐dried in a vacuum environment (13.33 Pa absolute pressure) at a condenser temperature of −48°C after being frozen for 4 h at −18°C in a freezer (Arçelik 5430). The vacuum level, or absolute pressure, was maintained at 13.33 Pa. The freeze dryer was set at a heating plate temperature of 20°C and a condenser temperature of −48°C ± 2°C for a duration of 9 h. The code of the freeze‐dried sample is FD.

The sun‐drying trials were conducted in July and August (normal harvest season) in Malatya, Türkiye, at the geographical coordinates 38° 21′ N and 38° 20′ E. The FB, approximately 150 g each, were evenly distributed onto trays measuring 0.45 × 0.45 m^2^ and promptly placed in the sunlight. Each trial began at 8 a.m. and ended until 6 p.m. The average relative humidity was 24%, and the air temperature varied between 29 and 33°C during sun‐drying. The code of the sun‐dried sample is SD.

A laboratory tray dryer (Eksis Mach.) was used for the convective drying (CD) tests. The tray dryer consisted of a temperature controller, a centrifugal blower for air flow generation, an electric heater, and 10 trays measuring 0.20 × 0.20 m^2^. The dryer was run at 45°C for 6 h, 50°C for 5 h, and 55°C for 4 h, with an air velocity of 1.5 m/s. The samples weighing approximately 50 g FB were arranged in a single layer in the metal trays, and the drying process was initiated. The codes of the convectively dried samples at 45, 50, and 55°C are CD45, CD50, and CD55, respectively.

The experiments were conducted in a domestic microwave oven (Arcelik MD 592) at 350, 460, and 600 W for 8.0, 6.0, and 4.5 min, respectively. The glass plate was covered with approximately 30 g of FB in a thin layer and dried. The codes for the microwave‐dried samples at power levels of 350, 460, and 600 W are MD350, MD460, and MD600, respectively. Purple basil leaves dried by all methods were powdered with a blender (Bosch MKM6000), and the powder obtained was stored in capped glass bottles (+4°C) in the refrigerator until analysis.

### 2.3 Color measurement

The color values (L*, a*, and b*) of the samples were measured using a CIE LAB colorimeter (Konica Minolta Colorimeter CR‐5). A typical white tile was used to calibrate the calorimeter. The L* values indicate darkness (black = 0/ white = 100), the a* value represents redness (positive)/greenness (negative), and the b* value indicates yellowness (positive)/blueness (negative). The Δ*E* value, utilizing a FB as a reference ($L_{\mathrm{control}}$, $a_{\mathrm{control}}$, and $b_{\mathrm{control}}$), is defined according to the following equation:

(1) $$\Delta E=\sqrt{{\left(L_{\mathrm{sample}}-L_{\mathrm{control}}\right)}^{2}+{\left(a_{\mathrm{sample}}-a_{\mathrm{control}}\right)}^{2}+{\left(b_{\mathrm{sample}}-b_{\mathrm{control}}\right)}^{2}}$$

### 2.4 Determination of total phenolic content (TPC), antioxidant activity (AA), and total anthocyanin content (TAC)

Total phenolic content (TPC) of the samples was analyzed according to the Folin–Ciocalteu method as outlined by Singleton et al. (1999). A 0.5‐g sample and 25 mL with methanol containing 0.1% (v/v) HCl were mixed and vigorously vortexed. Following the extraction procedure, all samples were stored in a freezer at −18°C for 24 h. A 40‐µL methanolic extract was combined with 3.16 mL of water and 200 µL of the Folin–Ciocalteu reagent, followed by 5 min of vortexing. The solution was then mixed with 600 µL of 2% Na_2_CO_3_ (w/v) and allowed to rest for 5 min. The mixture was then incubated for 120 min at 20°C, again in the dark. A spectrophotometer (Shimadzu, UV‐1800) measured the absorbance at 765 nm. Using gallic acid as a reference, the outcomes were given as mg GAE/g dry weight (dw).

The antioxidant activity (AA) of purple basil leaves was investigated using the ABTS (2,20 ‐azino‐bis(3‐ethylbenzthiazoline‐6‐ sulphonic acid)) (Xu et al., 2009) and DPPH (2,2‐diphenyl‐1‐picrylhydrazyl) (Lucena et al., 2010) methods. For both methods, the extract was obtained by mixing 0.1 g of the sample with 50 mL of pure methanol and shaking vigorously. The ABTS solution was made by mixing 7.0 mM ABTS and 2.45 mM K_2_S_2_O_8_ and stored at ±22°C for 18 h in the dark. The resulting mixture was diluted with methanol to get an absorbance of 0.70 ± 0.02 at 30°C. The sample (100 µL) and ABTS (3900 µL) were mixed in a test tube, and the absorbance of the basil leaves was determined at 734 nm via the same spectrophotometer. For the DPPH assay, 3.9 mL of DPPH was combined with the extract solution (1 mL). The solution was allowed to stand at 20°C for 45 min, and the absorbance was measured at 515 nm using the same spectrophotometer. The AA for ABTS and DPPH methods was calculated as mg Trolox equivalent (TE)/g dw.

Total anthocyanin content (TAC) was calculated using the Dimitrovska et al. (2011)‐described methodology. A 0.5‐g sample was mixed with 5 mL of methanol and water (10:90). Then, 0.25 mL of the extract solution was added to 4.55 mL of methanol containing 1% (w/v) HCl. The solution was kept in the dark for 15 min and determined at 520 nm with the same spectrophotometer. The TAC was calculated as malvidin‐3‐glycoside/g dw (AE/g dw).

### 2.5 Determination of volatiles in the samples

Volatiles in the leaf samples were determined by the solventless solid‐phase microextraction (SPME) method as described by Gurkan and Hayaloglu (2023). The basil leaves (2.0 g fresh basil or 1.0 g dried powder basil leaves), 2 mL distilled water, and 10 µL of internal standard (50 ppm 2‐methyl‐3‐heptanone) were mixed in a 15 mL crimp‐cap vial. The sample vial was continuously stirred at 600 rpm in combination with a heater and kept at 45°C for 30 min. Afterward, an SPME fiber (2 cm 50/30 µm DVB/Carboxen/PDMS; Supelco) was introduced into the headspace of the vial to capture all volatile compounds and maintained in this state for 30 min. After the absorption phase, the fiber was inserted into gas chromatography–mass spectrometry (GC–MS) (Shimadzu Corp.) for the desorption of the volatile components for 3 min in the column. A capillary column (DB‐Wax, 60 m × 0.25 mm × 0.25 µm; J&W Scientific) was used to separate the volatile compounds, and comparison of the retention index (RI) of the volatiles allowed for their identification and mass spectra on the DB‐Wax column using a commercial database of spectra. The NIST and Wiley libraries were used to match the mass spectra of each compound, and 35 authentic standards (Sigma‐Aldrich) for volatiles under the same chromatographic conditions were used for confirmation. RIs were calculated using a mixture of C_10_–C_26_ *n*‐alkenes. The fiber was adjusted to 3.0 scale units (indicated on the injection apparatus) for each run. The volatile compounds were desorbed using a Shimadzu GC‐2010 gas chromatograph coupled to a QP‐2010 mass spectrometer (Shimadzu Corp.) in splitless mode. Throughout the desorption procedure, the fiber remained in the injector at 250°C for 3 min, whereas helium was used as the carrier gas at a flow rate of 1.0 mL/min. The oven temperature was initially maintained at 40°C for 2 min during the desorption phase. The temperature was then increased by 5°C/min until it reached 70°C, where it was maintained for 1 min. Subsequently, the temperature was increased by 10°C/min up to 240°C, resulting in a total run time of 30 min. The volatile compounds were quantified utilizing the internal standard (semi‐quantification method) with 2‐methyl‐3‐heptanone as µg/g dw (Keskin et al., 2021). Quantification was performed using 2‐methyl‐3‐heptanone as an internal standard. The response factors were established by calculating the intensity ratio of each compound in comparison to 2‐methyl‐3‐heptanone, and the peak area ratios were set according to the specific response factor for each volatile. As a result, quantification was determined using provided Equation (2). Following that, the averages and standard deviations were determined with triplicate GC analyses:

(2) $$\;C_{I}=\frac{A_{I}}{A_{\mathrm{STD}}}\times C_{\mathrm{STD}}\times \mathrm{RF}\times \mathrm{CF}$$

where $C_{I}$ is the concentration of volatile compound; $A_{I}$ is the peak area of volatile compound; $A_{\mathrm{STD}}$ is the peak area of internal standard; $C_{\mathrm{STD}}$ is the concentration of internal standard; RF is the response factor; and CF is the calculation factor.

### 2.6 Determination of essential oil (EO) in the samples

The hydrodistillation of the EO from the purple basil leaves was assessed using the modified hydrodistillation method outlined by Teixeira et al. (2013). The Clevenger apparatus was attached to a 2‐L conical flask containing 100 g of fresh/50 g of dried leaves for the isolation of EOs. A volume of 500 mL of the flask was filled with pure water and heated until boiling. After 5‐h distillation, the EO was condensed by the Clevenger apparatus in a measuring cylinder section. The condensed EO and the aqueous layer were separated. The resulting oil was stored in a refrigerator at −18°C until it was analyzed by GC–MS. Identification of volatiles and chromatographic parameters was the same methodology as given in Section 2.5. Volatile contents in EO of purple basil leaves were presented as mean values of percentage of the total EO.

### 2.7 Statistical analysis

The drying trials and analyses were conducted three times and given as mean ± standard deviation. Statistical analysis was performed with SPSS version 20.0 software (SPSS Inc.). Duncan's multiple testing was used (*p *< 0.05) to compare means. GraphPad Prism 9 software was used to perform a one‐way ANOVA analysis of the information obtained. The significance level was indicated by the notations * *p *< 0.05, ** *p *< 0.01, and *** *p *< 0.001. The heat map analysis of the data was used to assess the impacts of the various drying processes on the volatiles and EO compounds and was carried out with the online software R‐Studio (https://www.r‐project.org).

## 3 RESULTS AND DISCUSSION

### 3.1 Color values (L*, a*, b*, and Δ*E*) of the purple basil leaves

The measurement of color is an important quality characteristic that indicates the quality and sensory evaluation of the dried samples (Quek et al., 2007). The color values of the purple basil leaves are presented in Table 1. One of the superior features of geographically registered Arapgir purple basil leaves is their dark purple color compared to many other basils. The drying conditions and methods had a statistical influence on the color and Δ*E* values of the purple basil leaves (*p *< 0.05). The L*, a*, b*, and Δ*E* values of the purple basil leaves varied among 21.50–26.80, 2.71–5.49, −0.26 to 5.17, and 0.00–6.03, respectively, showing that increasing the drying temperature had a notable effect on the L*, a*, b*, and Δ*E* values of the purple basil leaves (*p *< 0.05). The L* values of the samples decreased as the microwave power increased during drying (*p* < 0.05).

**TABLE 1:** Color values of Arapgir purple basil (*Ocimum basilicum* L.) leaves in fresh and dried at different conditions.

Sample	L*	a*	b*	Δ*E*
FB	25.15 ± 0.50^ab^	5.49 ± 0.30^a^	−0.26 ± 0.04^d^	0
FD	26.09 ± 0.04^a^	2.80 ± 0.04^b,c^	2.57 ± 0.10^a^	4.05 ± 0.29^b^
SD	23.00 ± 0.18^c^	3.55 ± 0.07^b^	2.53 ± 0.24^a^	4.04 ± 0.09^b^
CD45	23.53 ± 0.11^c,p^	3.15 ± 0.03^b,p^	1.39 ± 0.03^b,p^	3.33 ± 0.07^c,r^
CD50	23.21 ± 0.24^c,p^	3.51 ± 0.04^b,p^	1.15 ± 0.05^b,p^	3.17 ± 0.24^c,r^
CD55	22.69 ± 0.13^c,p^	2.71 ± 0.05^bc,r^	0.94 ± 0.03^c,r^	3.94 ± 0.24^a,p^
MD350	26.17 ± 0.75^a,x^	2.95 ± 0.21^bc,y^	2.80 ± 0.20^a,x^	4.25 ± 0.37^ab,y^
MD460	24.07 ± 0.21^bc,x^	3.05 ± 0.12^b,y^	2.17 ± 0.32^a,xy^	3.58 ± 0.17^bc,y^
MD600	21.50 ± 0.95^d,z^	3.44 ± 0.41^b,x^	1.60 ± 0.21^b,y^	4.88 ± 0.19^a,x^

*Note*: Data represent the mean values and standard deviations. FB, fresh sample; FD, freeze‐dried sample; SD, sun‐dried sample; CD45, convection‐dried sample at 45°C; CD50, convection‐dried sample at 50°C; CD55, convection‐dried sample at 55°C; MD350, microwave‐dried sample at 350 W; MD460, microwave‐dried sample at 460 W; MD600, microwave‐dried sample at 600 W.

^a–d^Statistical significance between FB and basil leaves dried by different methods (*p *< 0.05).

^p–s^Statistical significance between convection‐dried samples at different temperatures (*p *< 0.05).

^x–z^Statistical significance between microwave‐dried samples at different microwave power (*p *< 0.05).

The a* and b* values of the microwave‐dried purple basil leaves show no significant changes (*p* > 0.05). It is hypothesized that the increase in temperature with increasing microwave power could lead to pigment degradation or non‐enzymatic browning reactions that could cause blackening of the microwave‐dried samples. Chaves et al. (2022) dried purple basil leaves by CD (40–70°C), and the L* and a* values of the leaves exhibited a similar manner as in current research. Alibas et al. (2021) found that the L* values of basil leaves (*O. basilicum* L. var. “Sweet”) had higher values when dried naturally and convectively at 50°C than when dried by the microwave drying method at 100, 300, 500, 700, and 900 W. They observed that the green of the leaves disappeared at 100 and 300 W, whereas redness appeared. The authors also reported that at other microwave powers (500, 700, and 900 W), the a* values of the samples were very high and almost matched the color of fresh samples, and the b* values of the samples were also extremely high. In another study, the drying properties, volatiles, and color properties of holly basil (*Ocimum tenuiflorum* L.) were investigated in a microwave oven at 300, 450, 600, and 800 W and in a freeze dryer (Imaizumi et al., 2021). They found that the L* value of freeze‐dried and fresh leaves was the same and that the L* values of microwave‐dried leaves were lower than those of freeze‐dried and fresh samples, which is in accordance with the findings of the current research. In addition, drying led to an increase in a* and a decrease in b* values for both methods. As can be seen from the literature, different color values were observed in the studies because the plant *O. basilicum* L. has purple‐ or green‐colored varieties and the plant was dried at different temperatures, and the drying conditions affect the color values in a detectable manner.

### 3.2 The total phenolic content (TPC) of the purple basil leaves

**FIGURE 1:**
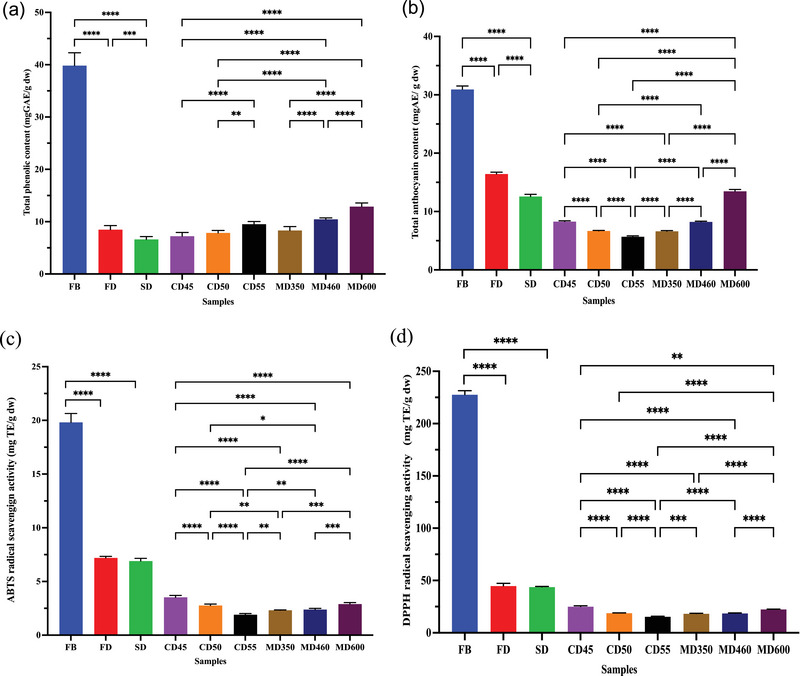
(a) Total phenolic content, (b) total anthocyanin content (TAC), (c) ABTS, and (d) DPPH radical scavenging activity of the samples (FB, fresh sample; FD, freeze‐dried sample; SD, sun‐dried sample; CD45, convection‐dried sample at 45°C; CD50, convection‐dried sample at 50°C; CD55, convection‐dried sample at 55°C; MD350, microwave‐dried sample at 350 W; MD460, microwave‐dried sample at 460 W; MD600, microwave‐dried sample at 600 W) (*p < 0.05, **p < 0.01, ***p < 0.001, ****p < 0.0001) (mean ± SD).

The TPC value of the samples is shown in Figure 1. Among the samples, FB had the highest TPC value (39.83 mg GAE/g dw), followed by 10.45 and 12.89 mg GAE/g dw for MD460 and MD600, respectively (*p* < 0.05). Our findings are consistent with previous studies in which basil leaf samples showed a decrease in phenolic content during microwave drying, sun‐drying, and convection drying, as noted by Chaves et al. (2022). The data from our study indicate that an increase in drying temperature and microwave power resulted in a significant increase in the TPC of purple basil leaves (*p *< 0.0001) (Figure 1). Compared to FB, the lowest total phenolic loss was observed at MD600. In addition, increasing the drying temperature and microwave power improved the preservation of TPC of the samples by an average of 16.28% and 27.98%, respectively. It was found that the TPC of CD55 (9.53 mg GAE/g dw) and MD600 (12.89 mg GAE/g dw) was higher than that of the other convection‐ and microwave‐dried samples. The reason for the high TPC protection when the drying temperature and microwave power were increased may be attributed to the fact that the drying period of the purple basil leaves was shortened and the samples were exposed to the destructive effect of temperature for a shorter time. Previous studies on kiwi (Izli et al., 2014), pear (Santos et al., 2014), and apple (Vega‐Ga´lvez et al., 2012) have also shown that the phenolic compounds were less exposed to the thermal effects due to the short drying time. Similar results were found for the TPC of oregano samples obtained by vacuum microwave drying (Jaloszynski et al., 2008). Busic et al. (2014) determined that the samples of *O. basilicum* L. obtained by freeze, CO_2_, and oven drying (OD) varied between 9.47 and 35.01 mg GAE/g dw. In addition, the TPC of oven‐dried samples was found to be 11 mg GAE/g dw. Similarly, in our study, comparable results were obtained from convection‐dried samples, which ranged from 7.21 to 9.53 mg GAE/g dw.

### 3.3 The total anthocyanin content (TAC) of the purple basil leaves

Anthocyanins are of crucial importance for assessing the antioxidant potential of foods. They are bioactive compounds that decompose to a high degree. Figure 1 shows the TAC of purple basil leaves. The TAC in FB amounted to 30.94 mg AE/g dw. After drying, FD had the highest TAC at 16.45 mg AE/g dw, whereas CD55 had the lowest TAC at 5.70 mg AE/g dw. The TAC decreased dramatically as the drying temperature increased, whereas the increase in microwave power led to a remarkable rise in the TAC (*p *< 0.0001) (Figure 1). Kaba et al. (2023) explained that the processes by which anthocyanins are degraded are complex, and in some cases, increasing temperature may have the unexpected effect of promoting color stability. This could be because anthocyanins have a dynamic chemical structure and complicated processes and transform into different substances depending on the temperature.

In the literature, the TAC for 15 different cultivars of *O. basilicum* L. was reported to be 0.48–8.74 mg/g dw (Kwee & Niemeyer, 2011). They reported that the highest TAC was observed in dark, purple‐colored cultivars. In another study, anthocyanin levels were between 7.55 and 16.6 mg/g dw in Rubin and Purple Ruffles cultivars, respectively (Flanigan & Niemeyer, 2014). McCance et al. (2016) calculated the TAC of the varieties Sweet Petra Dark Red and Purple Ruffles to be 2.07 and 9.72 mg/g dw, respectively. The total monomeric anthocyanins were calculated as 1.74, 4.45, and 10.91 mg/g dw0 for the oven‐dried (80°C), microwave‐dried (90 W for 2 min), and shade‐dried (room temperature) samples of purple basil leaf (Gulhan et al., 2022). In our study, the TAC content of the microwave‐dried samples was in the range of 6.64–13.49 mg AE/g dw, whereas the convection‐dried samples were in the range of 5.70–8.30 mg AE/g dw. The fact that anthocyanin levels were higher in our study compared to the study by Gulhan et al. (2022) is probably due to the distinct, more intense purple color of the Arapgir purple basil.

### 3.4 Antioxidant activity (AA) of the purple basil leaves

Many herbs and spices belonging to the Lamiaceae family have antioxidant properties. The genera belonging to this family have an antioxidant and antimicrobial effect, mainly because they contain terpene compounds (mono‐, di‐, and triterpenes), flavonoids, and phenolic acids (Fadıloğlu & Emir‐Çoban, 2018). AA of purple leaves was performed using two different methods, as shown in Figure 1. The highest ABTS and DPPH scavenging activity of purple basil leaves were obtained for FB with 19.81 and 227.72 mg TE/g dw, respectively.

The ABTS and DPPH values of the dried samples ranged from 1.89 to 7.19 and 15.48 to 44.89 mg TE/g dw, respectively. These two techniques are based on different mechanisms and do not evaluate the same radical in the sample. Therefore, it is not possible to compare the methods as the AA values of each sample are different. In addition, the chemicals have synergistic or inhibitory effects on each other (Altay et al., 2024).

Similar to anthocyanin results, ABTS and DPPH values of the samples decreased significantly with increasing drying temperature (*p *< 0.0001), whereas increasing microwave power led to a significant increase in ABTS (*p* < 0.001) and DPPH levels (*p *< 0.0001) (Figure 1). FD and SD exhibited the highest ABTS and DPPH values, whereas the CD55 had the lowest levels of both ABTS and DPPH. A sustainable processing method for preserving phenolic substances and AA is freeze drying.

The AA value of FD and SD was higher than that of convection‐ and microwave‐dried purple basil leaves. Lim and Murtijaya (2007) also found that the AA values of sun‐dried plant material (*Phyllanthus amarus*) were higher than those of microwave‐ and oven‐dried samples and explained this as follows: Destructive enzymes such as polyphenol oxidase are not immediately inactivated by sun‐drying, and the phenolic compounds are inactivated before the samples are completely dried. In addition, microwave drying has been reported to lead to faster inactivation of some polyphenols and greater loss of TPC and AA in oven‐dried samples because of the extended exposure to heat.

Chan et al. (2012) determined the AA of eight different plant samples from the Lamiaceae family, including *O. basilicum* L., obtained by OD at 50 and 80°C and OD at 50°C with microwave pretreatment (MP). The researchers reported that the AA of the oven‐dried samples at 50 and 80°C decreased by 88% and 95%, respectively, and the AA of the OD at 50°C with MP decreased by 73%. These results were consistent with our findings. The AA of CD45, CD50, and CD55 decreased with elevating the drying temperature, and the loss of AA compared to the fresh sample was found to be 89.0%, 91.7%, and 93.2%, respectively. Sharma et al. (2018) investigated the AA of basil leaves obtained by microwave (7.5 min), sun (70 h), and tray drying (45–55°C, 10 h) and found that microwave‐dried samples had higher AA, which is not in agreement with our results. They reported that microwave drying required a shorter period of time compared to drying in the oven and in the sun. They explained this situation by the fact that microwave drying takes less time compared to other drying methods. In our study, microwave drying resulted in similar values to CD but lower values than sun‐drying. It is assumed that this is due to the fact that the purple basil leaves were dried at higher microwave powers than in the study by Sharma et al. (2018).

### 3.5 Volatile compounds of the purple basil leaves

The volatiles found in purple basil leaves, including terpenes, alcohols, aldehydes, ketones, and aromatic compounds, were determined by GC–MS (Table 2). Major compounds in fresh and dried basil leaves are linalool, 1,8‐cineole, methyl cinnamate, and dl‐limonene.

**TABLE 2:** Volatile compounds of Arapgir purple basil (*Ocimum basilicum* L.) leaves in fresh and dried at different conditions (µg/g dw).

Name	RI^dbw^	RI^lit^	FB	FD	SD	CD45	CD50	CD55	MD350	MD460	MD600
**Monoterpene hydrocarbons**											
α‐Pinene	1021	1023^a^	46.84 ± 1.24^d^	67.82 ± 1.12^g^	7.61 ± 0.28^a^	56.68 ± 2.17^f,r^	96.77 ± 0.91 ^h,s^	51.88 ± 0.05^e,p^	13.63 ± 0.35^b^ ^,y^	18.76 ± 1.49^c,z^	8.75 ± 0.31^a,x^
Camphene	1066	1040^b^	N.D.	1.07 ± 0.05^d^	0.55 ± 0.08^b^	1.92 ± 0.12^e,p^	6.28 ± 0.00^f,s^	2.08 ± 0.03^e,r^	0.79 ± 0.14^c,y^	0.34 ± 0.05^a,x^	0.22 ± 0.02^a,x^
α‐Terpinene	1185	1167^c^	N.D.	3.6 ± 0.30^e^	2.08 ± 0.07^c^	2.56 ± 0.05^d,p^	15.06 ± 0.03^g^ ^,s^	5.39 ± 0.01^f,r^	2.70 ± 0.05^d,z^	1.20 ± 0.47^b^ ^,y^	0.48 ± 0.02^a,x^
dl‐Limonene	1189	1187^d^	291.47 ± 2.51^h^	49.57 ± 2.25^d^	17.17 ± 1.96^b^	20.18 ± 0.33^b^ ^,p^	91.18 ± 0.84^g^ ^,s^	56.13 ± 1.09^e,r^	45.52 ± 0.20^c,y^	86.02 ± 1.02^f,z^	7.01 ± 0.60^a,x^
(*Z*)‐β‐Ocimene	1236	1238^e^	N.D.	5.54 ± 0.03^b^	2.88 ± 0.07^a^	6.40 ± 0.43^b^ ^,r^	12.44 ± 1.19^c,s^	5.65 ± 0.02^b^ ^,p^	N.D.	N.D.	N.D.
γ‐Terpinene	1246	1240^a^	4.60 ± 0.10^b^	5.66 ± 0.51^c^	5.69 ± 0.00^c^	N.D.	N.D.	N.D.	2.62 ± 0.00^a,x^	2.81 ± 0.00^a,x^	N.D.
**Total**			342.91	133.26	35.98	87.74	221.73	121.13	65.26	109.13	16.46
**Oxygenated monoterpenes**											
α‐Phellandrene	1025	–	3.00 ± 0.09^d^	1.91 ± 0.05^c^	1.05 ± 0.10^a,^ ^b^	1.79 ± 0.02^c,p^	17.21 ± 0.00^e,r^	N.D.	1.12 ± 0.30^b^ ^,x^	0.79 ± 0.19^a,x^	0.90 ± 0.08^ab,x^
β‐Pinene	1102	1130^a^	68.21 ± 9.08^d^	52.20 ± 1.69^c^	12.66 ± 0.92^a^	33.05 ± 0.68^b^ ^,p^	57.48 ± 3.66^c,s^	25.98 ± 0.86^b^ ^,r^	14.64 ± 1.21^a,z^	6.45 ± 0.31^a,x^	11.28 ± 1.74^a,y^
β‐Myrcene	1149	1143^d^	106.75 ± 3.80^f^	57.14 ± 2.42^d^	18.6 ± 0.89^b^	37.87 ± 0.80^c,p^	94.42 ± 3.19^e,s^	52.70 ± 1.75^d,r^	13.76 ± 1.34^ab,y^	16.74 ± 1.58^b^ ^,z^	11.11 ± 1.19^a,x^
1,8‐Cineol	1212	1209^a^	816.16 ± 23.90^e^	279.74 ± 4.89^d^	93.66 ± 2.98^b^	91.00 ± 0.92^b^ ^,p^	171.22 ± 2.54^c,s^	95.30 ± 3.71^b^ ^,r^	58.01 ± 0.28^a,z^	56.77 ± 0.93^a,y^	45.15 ± 0.05^a,x^
p‐Cymene	1272	1268^a^	5.96 ± 0.40^b^	N.D.	4.08 ± 0.18^a^	5.21 ± 0.07^b^ ^,p^	19.52 ± 0.95^c,r^	5.30 ± 0.01^b^ ^,p^	N.D.	N.D.	N.D.
α‐Terpinolene	1283	1280^a^	6.26 ± 0.30^c^	4.23 ± 0.44^b^	N.D.	7.07 ± 0.11^d,p^	23.04 ± 0.83^f,s^	8.94 ± 0.10^e,r^	0.87 ± 0.05^a,x^	N.D.	0.82 ± 0.00^a,x^
(*E*)‐Sabinene hydrate	1477	1463^f^	83.17 ± 0.40^f^	20.31 ± 1.34^e^	5.31 ± 0.36^c^	N.D.	7.58 ± 0.52^d,r^	4.70 ± 0.14^c,p^	1.02 ± 0.05^ab,x^	1.69 ± 0.03^b^ ^,y^	1.06 ± 0.09^ab,x^
(*Z*)‐Linalool oxide	1487	1430^g^	12.06 ± 0.36^f^	10.33 ± 0.70^e^	4.84 ± 0.06^c^	6.40 ± 0.39^d,s^	4.22 ± 0.04^c,r^	2.71 ± 0.06^b^ ^,p^	0.42 ± 0.14^a,x^	0.54 ± 0.05^a,x^	0.38 ± 0.00^a,x^
Camphor	1544	1538^a^	N.D.	N.D.	N.D.	5.30 ± 0.56^a,p^	N.D.	N.D.	N.D.	N.D.	N.D.
Linalool	1549	1555^a^	1176.09 ± 23.31^e^	395.22 ± 6.21^d^	N.D.	259.06 ± 4.17^c,s^	245.79 ± 2.27^c,r^	166.3 ± 2.04^b^ ^,p^	81.19 ± 7.72^a,x^	83.15 ± 4.81^a,y^	N.D.
Linalool‐l	1550	–	N.D.	109.89 ± 2.09^d^	176.58 ± 1.4^e^	N.D.	N.D.	30.15 ± 0.00^b^ ^,p^	9.52 ± 0.00^a,x^	N.D.	51.54 ± 1.51^c,y^
α‐Guaiene	1607	1588^c^	53.02 ± 1.52^e^	12.42 ± 1.05^c^	2.30 ± 0.85^a^	11.03 ± 0.77^c,r^	16.22 ± 0.07^d,s^	8.23 ± 0.10^b^ ^,p^	2.70 ± 0.66^a,x^	2.06 ± 0.3^a,x^	2.21 ± 0.06^a,x^
p‐Menth‐1‐en‐8‐ol	1688	–	27.59 ± 4.49^d^	8.97 ± 0.87^c^	1.93 ± 0.10^ab^	4.78 ± 0.83^b^ ^,r^	4.00 ± 0.14^ab,r^	1.76 ± 0.09^ab,p^	0.23 ± 0.00^a^	0.45 ± 0.09^a^	0.21 ± 0.09^a^
α‐Terpineol	1712	1695^h^	102.94 ± 9.58^e^	22.38 ± 0.62^d^	7.34 ± 0.66^ab^	12.92 ± 0.05^bc,r^	15.90 ± 0.52^cd,s^	11.53 ± 1.05^bc,p^	2.43 ± 0.81^a,y^	2.32 ± 0.21^a,y^	1.12 ± 0.00^a,x^
Borneol‐l	1721	1698^g^	N.D.	N.D.	N.D.	2.96 ± 0.10^a,r^	6.74 ± 2.15^b^ ^,s^	2.09 ± 0.11^a,p^	N.D.	N.D.	N.D.
Geraniol	1853	1837^a^	N.D.	N.D.	N.D.	N.D.	0.55 ± 0.00^a,p^	N.D.	N.D.	N.D.	N.D.
**Total**			2461.21	974.74	328.35	478.44	683.89	415.69	185.91	170.96	125.78
**Sesquiterpene hydrocarbons**											
α‐Cubebene	1465	1463^a^	23.24 ± 1.23^f^	5.53 ± 0.00^d^	0.96 ± 0.08^ab^	N.D.	7.29 ± 0.09^e,r^	3.21 ± 0.20^c,p^	1.19 ± 0.18^b^ ^,y^	0.67 ± 0.08^ab,x^	0.42 ± 0.01^ab,x^
α‐Copaene	1504	1517^b^	33.96 ± 0.92^e^	5.57 ± 0.96^c^	1.54 ± 0.08^a^	3.33 ± 0.44^b^ ^,p^	11.42 ± 0.45^d,s^	6.42 ± 0.15^c,r^	1.58 ± 0.01^a,y^	0.95 ± 0.28^a,x^	0.56 ± 0.01^a,x^
β‐Bourbonene	1536	1541^i^	29.99 ± 4.07^d^	2.02 ± 0.46^a^	N.D.	N.D.	10.11 ± 0.28^c,r^	6.45 ± 0.04^b^ ^,p^	1.03 ± 0.00^a,y^	N.D.	0.51 ± 0.01^a,x^
*(E)‐*α‐*trans*‐Bergamotene	1594	1595^h^	203.88 ± 6.42^f^	47.54 ± 1.03^d^	9.92 ± 0.03^ab^	29.30 ± 0.48^c,p^	52.76 ± 1.07^e,r^	28.82 ± 0.49^c,p^	6.39 ± 0.25^ab,z^	5.86 ± 0.07^ab,y^	4.30 ± 0.26^a,x^
β‐Elemene	1601	1587^b^	165.10 ± 3.38^f^	38.81 ± 1.56^e^	5.44 ± 0.49^a^	14.75 ± 0.56^b^ ^,p^	28.19 ± 0.45^d,s^	24.41 ± 1.24^c,r^	5.74 ± 0.93^a,y^	3.77 ± 0.6^a,x^	N.D.
(*E*)‐β‐Caryophyllene	1619	1649^i^	21.66 ± 3.54^c^	12.27 ± 1.19^b^	N.D.	1.66 ± 0.23^a,r^	3.06 ± 0.34^a,pr^	2.31 ± 0.05^a,p^	N.D.	N.D.	N.D.
Aromadendrene	1626	1643^c^	12.10 ± 1.27^d^	0.83 ± 0.00^ab^	1.00 ± 0.00^ab^	2.45 ± 0.22^c,r^	2.40 ± 0.29^c,r^	1.18 ± 0.02^b^ ^,p^	N.D.	N.D.	N.D.
β‐Cedrene	1627	–	13.67 ± 1.58^b^	0.27 ± 0.00^a^	N.D.	N.D.	N.D.	N.D.	N.D.	N.D.	0.27 ± 0.00^a,x^
β‐Farnesene	1669	1671^h^	31.42 ± 3.61^e^	13.39 ± 0.07^cd^	1.88 ± 0.01^a^	10.90 ± 0.12^c,r^	15.29 ± 0.28^d,s^	6.86 ± 0.37^b^ ^,p^	1.42 ± 0.05^a,y^	0.75 ± 0.07^a,x^	0.55 ± 0.11^a,x^
α‐Humulene	1694	1681^h^	76.93 ± 5.89^e^	27.78 ± 0.47^d^	4.47 ± 0.59^b^	14.09 ± 0.69^c,p^	27.79 ± 0.28^d,r^	15.06 ± 0.73^c,p^	5.94 ± 0.11^b^ ^,z^	3.72 ± 0.57^a,y^	2.10 ± 0.03^a,x^
Valencene	1725	1718^i^	1.42 ± 0.11^d^	N.D.	N.D.	N.D.	1.25 ± 0.17^c,r^	0.80 ± 0.03^b^ ^,p^	N.D.	N.D.	0.25 ± 0.00^a,x^
Germacrene D	1733	1724^e^	244.29 ± 10.45^c^	17.47 ± 1.41^b^	5.20 ± 0.24^a^	7.83 ± 0.18^a,p^	25.10 ± 0.49^b^ ^,s^	21.53 ± 0.35^b^ ^,r^	2.51 ± 0.35^a,y^	2.89 ± 0.25^a,y^	1.33 ± 0.04^a,x^
α‐Bulnesene	1739	–	3.22 ± 0.42^c^	0.68 ± 0.08^a^	1.54 ± 0.03^b^	4.48 ± 0.04^d,p^	8.46 ± 0.00^e,r^	N.D.	N.D.	1.26 ± 0.07^b^ ^,y^	0.55 ± 0.01^a,x^
β‐Selinene	1752	1707^i^	18.56 ± 1.27^b^	N.D.	N.D.	N.D.	N.D.	N.D.	0.61 ± 0.12^a,x^	N.D.	N.D.
Bicyclogermacrene	1761	–	74.28 ± 5.10^e^	6.12 ± 0.63^c^	1.74 ± 0.22^a,^ ^b^	5.07 ± 0.95^bc,p^	14.68 ± 0.07^d,s^	7.29 ± 0.05^c,r^	1.87 ± 0.17^ab,y^	1.39 ± 0.28^ab,y^	0.64 ± 0.05^a,x^
γ‐Cadinene	1784	1749^e^	21.40 ± 2.90^e^	7.87 ± 1.43^b^	2.18 ± 0.14^a^	15.60 ± 0.28^c,r^	19.05 ± 1.54^d,s^	1.47 ± 0.06^a,p^	3.63 ± 0.54^a,z^	2.13 ± 0.07^a,y^	1.02 ± 0.05^a,x^
α‐Muurolene	1813	–	N.D.	N.D.	N.D.	N.D.	0.98 ± 0.06^b^ ^,r^	0.38 ± 0.01^a,p^	N.D.	N.D.	N.D.
(*Z*)‐Calamene	1862	–	10.48 ± 2.30^b^	N.D.	N.D.	N.D.	N.D.	N.D.	0.39 ± 0.00^a,x^	N.D.	N.D.
**Total**			985.60	186.15	35.87	109.46	227.83	126.19	32.30	23.39	12.50
**Oxygenated sesquiterpene**											
Caryophyllene	1622	1608^g^	52.80 ± 1.90^e^	N.D.	N.D.	N.D.	8.08 ± 0.13^d,r^	3.74 ± 0.13^c,p^	1.77 ± 0.11^b^	0.22 ± 0.12^a,x^	N.D.
α‐epi‐Muurolol	2188	–	51.62 ± 3.19^c^	3.55 ± 0.18^b^	0.76 ± 0.05^a^	3.06 ± 0.09^ab,p^	3.39 ± 0.34^b^ ^,p^	3.07 ± 0.30^ab,p^	0.51 ± 0.02^a,x^	0.65 ± 0.08^a,z^	0.52 ± 0.00^a,y^
**Total**			108.51	5.56	2.81	8.44	11.47	8.42	2.28	2.19	1.13
**Alcohols**											
1‐Penten‐3‐ol	1159	1155^f^	N.D.	2.27 ± 0.10^e^	1.37 ± 0.12^d^	2.75 ± 0.21^f,r^	0.74 ± 0.00^c,p^	3.98 ± 0.00^g^ ^,s^	N.D.	0.26 ± 0.02^b^ ^,y^	0.15 ± 0.06^ab,x^
2‐Penten‐1‐ol	1327	1313^a^	2.46 ± 0.32^a^	N.D.	N.D.	2.83 ± 0.02^b^ ^,p^	N.D.	N.D.	N.D.	N.D.	N.D.
1‐Hexanol	1359	1352^a^	8.77 ± 1.85^b^	1.03 ± 0.00^a^	N.D.	N.D.	N.D.	N.D.	N.D.	N.D.	N.D.
3‐Hexen‐1‐ol	1393	1388^a^	114.11 ± 6.97^c^	15.55 ± 0.46^b^	1.91 ± 0.06^a^	2.71 ± 0.12^a,p^	4.84 ± 0.08^a,r^	2.58 ± 0.12^a,p^	2.04 ± 0.58^a,y^	3.24 ± 0.05^a,z^	1.71 ± 0.80^a,x^
2‐Hexen‐1‐ol	1412	1390^i^	5.49 ± 0.21^a^	N.D.	N.D.	N.D.	N.D.	N.D.	N.D.	N.D.	N.D.
1‐Octen‐3‐ol	1452	1456^h^	265.27 ± 19.58^b^	13.68 ± 0.03^a^	3.11 ± 0.02^a^	5.41 ± 0.37^a,s^	2.70 ± 0.01^a,r^	1.55 ± 0.07^a,p^	N.D.	0.37 ± 0.02^a,y^	0.20 ± 0.04^a,x^
2‐Ethylhexanol	1493	–	48.12 ± 0.03^c^	0.77 ± 0.47^a^	0.47 ± 0.06^a^	3.00 ± 0.03^b,p^	N.D.	N.D.	N.D.	0.96 ± 0.11^a,y^	0.27 ± 0.07^a,x^
1‐Octanol	1562	1558^g^	2.23 ± 0.25^b^	4.19 ± 0.19^c^	1.68 ± 0.00^a^	4.63 ± 0.25^c,s^	2.94 ± 0.11^b,r^	1.55 ± 0.20^a,p^	N.D.	N.D.	N.D.
Benzyl alcohol	1894	1870^f^	N.D.	1.92 ± 0.14^b^	0.38 ± 0.01^a^	N.D.	0.47 ± 0.00^a,p^	N.D.	N.D.	3.37 ± 0.28^d,y^	2.68 ± 0.11^c,x^
Cubenol	2091	–	13.29 ± 1.48^b^	N.D.	N.D.	N.D.	0.73 ± 0.00^a,p^	0.82 ± 0.00^a,p^	0.34 ± 0.00^a,x^	N.D.	N.D.
**Total**			459.74	39.41	8.92	21.33	12.42	10.48	2.38	8.2	5.01
**Aldehydes and ketones**											
*Trans*‐β‐Ionone‐5,6‐epoxide	668	–	N.D.	N.D.	N.D.	1.18 ± 0.04^c,r^	0.84 ± 0.02^b,p^	1.52 ± 0.02^d,s^	1.60 ± 0.41^e,y^	N.D.	0.27 ± 0.02^a,x^
2‐Propanone	795	775^d^	40.67 ± 3.31^f^	26.96 ± 0.70^e^	10.28 ± 0.89^b^	7.60 ± 0.62^b,p^	44.06 ± 0.59^g^ ^,s^	22.06 ± 0.06^d,r^	14.56 ± 0.95^c,y^	41.00 ± 0.83^f,z^	2.31 ± 0.65^a,x^
2‐Methylbutanal	906	–	8.50 ± 0.56^c^	0.60 ± 0.06^a^	0.91 ± 0.08^a^	1.60 ± 0.33^b,r^	0.70 ± 0.16^a,p^	0.95 ± 0.03^a,pr^	0.49 ± 0.12^a,x^	0.40 ± 0.07^a,x^	0.53 ± 0.01^a,x^
3‐Methylbutanal	910	–	10.15 ± 0.04^d^	0.58 ± 0.08^a^	2.01 ± 0.03^c^	2.09 ± 0.54^c,s^	0.82 ± 0.03^a,p^	1.27 ± 0.09^b,r^	0.51 ± 0.13^a,x^	0.56 ± 0.05^a,x^	0.63 ± 0.12^a,x^
Hexanal	1080	1081^a^	4.04 ± 0.05^e^	4.21 ± 0.38^f^	1.66 ± 0.22^b^	3.42 ± 0.54^d,s^	2.23 ± 0.10^c,r^	1.26 ± 0.00^b,p^	0.56 ± 0.01^a,x^	1.19 ± 0.01^y^	0.59 ± 0.00^a,x^
2‐Hexanal	1225	1209^f^	222.06 ± 3.30^c^	8.94 ± 0.92^b^	1.12 ± 0.05^a^	6.49 ± 0.96^b,r^	1.81 ± 0.17^a,p^	1.60 ± 0.15^a,p^	N.D.	0.41 ± 0.05^a,x^	N.D.
Octanal	1294	1184^j^	N.D.	10.92 ± 0.81^e^	3.54 ± 0.14^b^	N.D.	7.97 ± 0.53^d,r^	5.83 ± 0.12^c,p^	1.18 ± 0.20^a,xy^	1.61 ± 0.11^a,y^	0.84 ± 0.08^a,x^
Nonanal	1399	1394^a^	N.D.	0.55 ± 0.04^ab^	1.73 ± 0.20^c^	4.85 ± 0.57^e,r^	2.44 ± 0.00^d,p^	4.28 ± 0.00^d,r^	0.76 ± 0.15^b,x^	1.39 ± 0.42^c,x^	1.54 ± 0.20^c,x^
2,4‐Heptadienal	1476	1483^g^	41.20 ± 2.09^e^	10.13 ± 0.84^c^	3.22 ± 0.11^a^	14.01 ± 0.04^ds^	6.55 ± 0.43^br^	4.70 ± 0.47^ap^	N.D.	N.D.	N.D.
3,5‐Octadien‐2‐one	1532	–	0.62 ± 0.35^ab^	14.47 ± 0.79^e^	5.55 ± 0.33^c^	11.12 ± 0.43^d,r^	1.35 ± 0.08^b,p^	1.38 ± 0.00^b,p^	0.19 ± 0.09^a,y^	0.24 ± 0.06^a,y^	0.05 ± 0.02^a,x^
β‐Ionone	1967	1912^a^	N.D.	1.00 ± 0.00^b^	0.43 ± 0.05^a^	3.16 ± 0.30^e,r^	1.62 ± 0.00^c,p^	1.94 ± 0.00^d,p^	N.D.	N.D.	N.D.
3‐Octanone	1256	1247^f^	11.14 ± 0.17^b^	0.73 ± 0.07^a^	N.D.	N.D.	N.D.	N.D.	N.D.	N.D.	N.D.
6‐Methyl‐5‐hepten‐2‐one	1343	1333^d^	5.26 ± 0.74^e^	4.34 ± 0.02^d^	1.61 ± 0.04^b^	2.34 ± 0.36^c,r^	1.38 ± 0.07^b,p^	2.70 ± 0.08^c,r^	0.50 ± 0.07^a,yz^	0.70 ± 0.08^a,z^	0.30 ± 0.04^a,xy^
**Total**			369.76	83.76	32.20	58.04	71.87	49.54	20.38	47.55	7.08
**Aromatic compounds**											
Xylene	1128	1030^j^	N.D.	0.74 ± 0.13^c^	0.44 ± 0.00^ab^	1.47 ± 0.01^d,s^	0.46 ± 0.07^ab,p^	0.83 ± 0.00^c,r^	0.39 ± 0.00^a,x^	0.54 ± 0.01^b,y^	N.D.
Styrene	1258	1253^d^	28.66 ± 0.24 g	3.58 ± 0.03^c^	1.29 ± 0.60^b^	6.83 ± 0.60^d,p^	10.90 ± 0.73^f,s^	7.35 ± 0.35^d,r^	4.10 ± 0.01^c,y^	9.08 ± 0.20^e,z^	0.28 ± 0.05^a,x^
Benzaldehyde	1543	1528^h^	34.41 ± 1.86^d^	38.29 ± 0.64^e^	15.69 ± 0.46^c^	11.29 ± 0.29^b,r^	14.64 ± 0.00^c,s^	10.21 ± 0.55^b,p^	3.33 ± 0.08^a,y^	3.59 ± 0.88^a,y^	2.01 ± 0.03^a,x^
Phenylethyl alcohol	1934	1906^i^	2.09 ± 0.21^b^	8.29 ± 1.77^d^	15.25 ± 0.40^e^	6.00 ± 0.67^c,s^	1.47 ± 0.20^ab,r^	0.76 ± 0.08^ab,p^	0.35 ± 0.00^a,x^	0.33 ± 0.09^a,x^	0.34 ± 0.00^a,x^
Methyleugenol	2017	2011^h^	N.D.	N.D.	0.75 ± 0.05^b^	N.D.	N.D.	6.74 ± 0.30^cp^	N.D.	0.45 ± 0.08^a,x^	0.53 ± 0.03^ab,y^
Methyl cinnamate	2104	2084^d^	637.65 ± 14.23^f^	162.61 ± 2.72^e^	66.00 ± 1.99^c^	92.60 ± 2.45^d,r^	91.18 ± 3.52^d,r^	81.11 ± 0.68^d,p^	34.08 ± 3.47^b,y^	40.62 ± 1.98^b,z^	13.20 ± 0.57^a,x^
Eugenol	2179	2172^h^	N.D.	29.26 ± 1.35^f^	N.D.	24.22 ± 0.31^e,s^	6.69 ± 0.18^d,r^	3.30 ± 0.00^c,p^	0.41 ± 0.19^a,x^	1.69 ± 0.08^b,x^	0.83 ± 0.01^ab,x^
Carvacrol	2196	2206^f^	N.D.	2.87 ± 0.55^a^	N.D.	N.D.	N.D.	N.D.	N.D.	N.D.	N.D.
**Total**			702.81	245.64	99.42	142.41	125.34	110.3	42.66	56.3	17.19

*Note*: Data represent the mean values and standard deviations from duplicate injections. FB, fresh sample; FD, freeze‐dried sample; SD, sun‐dried sample; CD45, convection‐dried sample at 45°C; CD50, convection‐dried sample at 50°C; CD55, convection‐dried sample at 55°C; MD350, microwave‐dried sample at 350 W; MD460, microwave‐dried sample at 460 W; MD600, microwave‐dried sample at 600 W. RI^dbw^: Retention indices calculated on DB‐WAX column using alkane standards. RI^lit^: Retention indices according to literature.

^a–i^Statistical significance between FB and basil leaves dried by different methods (*p *< 0.05).

^p–s^Statistical significance between convection‐dried samples at different temperatures (*p *< 0.05).

^x–z^Statistical significance between microwave dried samples at different microwave power (*p *< 0.05).

^a^ Roitman et al. (2011).

^b^Usami et al. (2013).

^c^Sonmezdag et al. (2018).

^d^Gurkan and Hayaloglu (2017).

^e^Jordan et al. (2017).

^f^Lee et al. (2005).

^g^Mahmoud et al. (2022).

^h^Kang et al. (2012).

^i^Sevindik et al. (2022).

^j^Gurkan and Hayaloglu (2023).

All purple basil leaves contained large amounts of terpene compounds. Monoterpene hydrocarbons were detected in high amounts (342.91 µg/g dw) in FB (Table 2). The lowest loss of monoterpene hydrocarbons was observed in the CD50 with 35.34% compared to the FB. It can also be observed that CD50 was the most effective method for preserving monoterpenes, whereas SD proved to be less favorable for the preservation of monoterpenes. The relative amount of some monoterpene hydrocarbons (camphene, α‐terpinene, and *cis*‐ocimene) was absent in FB. It can be recognized that drying increased the amount of α camphene (0.34–6.28 µg/g dw), α‐terpinene (1.20–15.06 µg/g dw), and *cis*‐ocimene (2.88–12.44 µg/g dw; Table 2).

Oxygenated monoterpenes such as linalool (1176.09 µg/g dw) and 1,8‐cineole (816.16 µg/g dw) were detected in high concentrations in FB. Among the dried samples, linalool (395.22 µg/g dw) and 1,8‐cineole (279.74 µg/g dw) were most abundant in FD. In addition, these compounds were better protected in FD compared to other drying methods. When convection‐ and microwave‐dried samples are compared within themselves, the amounts of oxygenated monoterpene compounds were higher in CD50 (683.89 µg/g dw) and MD350 (185.91 µg/g dw). The possible reason for this is the degradation of the volatile oxygenated monoterpene compounds due to the exposure of the samples to higher temperatures of 55°C and microwave powers of 460 and 600 W, as well as the prolonged exposure of the samples to temperatures of 45°C. A recent result of hot air–dried *Mentha haplocalyx* leaves showed a moderate decrease in terpenes such as cineole (1.018%, 0.893%, and 0.83%) and 1‐caryophyllene (2.614%, 2.486%, and 2.358%), with an increase in drying temperature from 35, 45, and 55°C, respectively (Guo et al., 2022). This study also reported that these volatile compounds are sensitive to heat and a high drying rate, and too high temperatures can cause damage or changes in the volatiles. The volatile sesquiterpene hydrocarbons such as germacrene (244.29 µg/g dw) and α‐*trans*‐bergamotene (203.88 µg/g dw) were most abundant in the FB. Increasing the microwave power decreased the amount of oxygenated sesquiterpenes in purple basil leaves at a significant level (*p *< 0.05). CD50 showed the least decline in both sesquiterpene hydrocarbons and oxygenated sesquiterpenes.

During drying, the amount of alcohols in purple basil leaves decreased. However, there are also alcohol compounds such as 1‐penten‐3‐ol and benzyl alcohol that are produced by the drying effect. A significant decrease in alcohol compounds of the samples was observed with the increase in drying temperature (*p *< 0.05). An increase in temperature is known to cause alcohol loss. In a study on *Capsella bursa‐pastoris* L., the impacts of drying techniques (hot air, microwave, vacuum, and freeze drying) on the aroma components of the samples were investigated (Zhang et al., 2016). They reported that, compared to fresh samples, the alcohol content decreased during hot air and microwave drying, whereas the alcohols were well preserved during vacuum and freeze drying. Similar results, the alcohol compounds, including 3‐hexen‐1‐ol, 1‐octen‐3‐ol, 2‐ethyl‐hexanol, 1‐octanol, and cubenol compounds of basil leaves, decreased with drying temperature were observed in our study. In addition, the best preservation method for alcohol compounds was found to be freeze drying. In general, aldehyde and ketone compounds of purple basil leaves decreased with drying. These compounds were found to be higher in CD50 and MD460 than in the other samples. This is thought to be due to the damage of these compounds caused by the exposure of the samples to higher temperatures of 55°C and a microwave power of 600 W, as well as the longer exposure to temperatures of 45°C and a microwave power of 350 W. *Trans*‐β‐Ionone‐5,6‐epoxide, octanal, nonanal, and β‐Ionone were not found in the FB, but these compounds were formed after drying.

The aromatic compound methyl cinnamate (637.65 µg/g dw) was most abundant in FB, whereas in dried samples, it was found in a range of 13.20–162.61 µg/g dw. Lee et al. (2005) reported that 1.28 mg/g methyl cinnamate was detected in commercially dried leaves of *O. basilicum* L. It was found that FD showed the lowest loss of methyl cinnamate compared to FB. In contrast with sun‐drying, microwave drying, and CD reduced the methyl cinnamate content more. In general, the lowest loss of volatile compounds was observed in the freeze‐drying method. Compared to other methods, freeze drying is performed at very low temperatures, which presumably reduces the loss of volatiles. It has been reported that volatile aromatic compounds are influenced by factors, such as atmospheric oxygen, humidity, temperature, and light (Cid‐Pérez et al., 2021).

**FIGURE 2:**
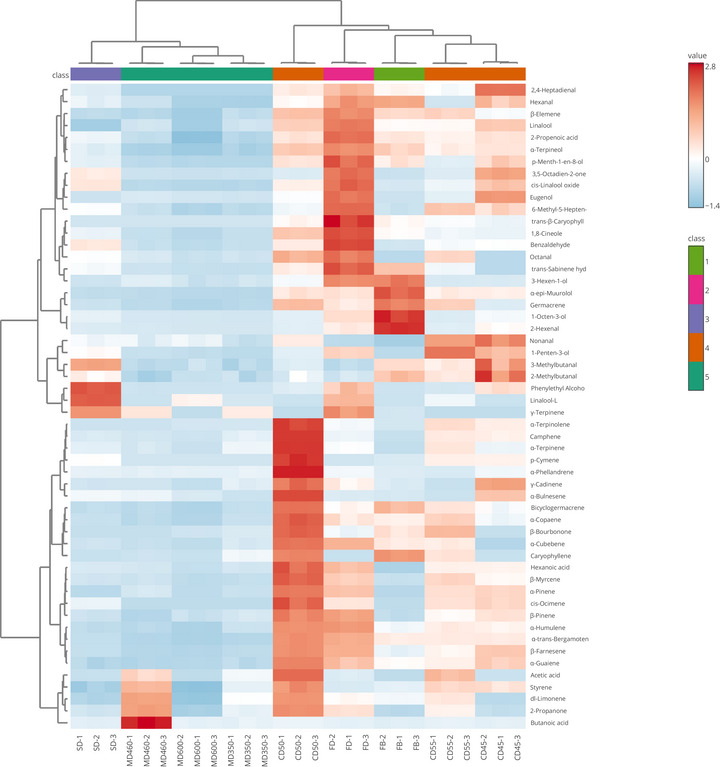
The heat map of volatiles from samples. Rows: volatile organic compounds identified; columns: basil leaves dried by different methods and fresh basil leaves. The color scale, ranging from low abundance (blue) to high abundance (red), may be inferred from the relative abundance in the display of heat maps (basil leaf codes are as follows: FB, fresh sample; FD, freeze‐dried sample; SD, sun‐dried sample; CD45, convection‐dried sample at 45°C; CD50, convection‐dried sample at 50°C; CD55, convection‐dried sample at 55°C; MD350, microwave‐dried sample at 350 W; MD460, microwave‐dried sample at 460 W; MD600, microwave‐dried sample at 600 W).

To better evaluate the diversity of volatiles, a heat mapping of the volatile data was carried out for visualization (Figure 2). The leaves were separated into two subclusters. The first cluster contains three sub‐groups including SD alone. MD350, 460, and 600 formed one group. The second cluster contains four subclusters, including CD50 alone, CD45–55 and FB, and FD formed one cluster as can be seen on the top of Figure 2. FB and FD showed similar profiles of volatile compounds both qualitatively and quantitatively, as shown in Figure 2. The volatile compounds include *trans*‐β‐caryophyllene, 1,8‐cineole, benzaldehyde, *trans*‐sabinene hydrate, β‐elemene, linalool, 2‐propenoic acid, p‐menth‐1‐en‐8‐ol, and eugenol, which were grouped, whereas FD exhibited by higher levels.

Similar to the volatile compounds detected in the current study, volatiles, such as linalool, 1,8‐cineole, and eugenol, have been reported to be commonly found in other basil drying studies (Diaz‐Maroto et al., 2004; Qu et al., 2019). 1,8‐Cineole and linalool were the major volatiles in the raw material and shade‐dried flowers, stems, and leaves of purple basil (Gurkan & Hayaloglu, 2023). The dried plant *O. basilicum* L. was found to contain more terpene compounds, such as β‐elemene, β‐caryophyllene, α‐bergamotene, germacrene‐d, and γ‐cadinene, in comparison to the fresh plant (Diaz‐Maroto et al., 2004). In addition, it was found that the above‐mentioned terpene compounds are generally present in higher amounts in fresh basil leaves. It is assumed that this could be due to the different geographical conditions, soil structure, and genetic structure of the plant.

In a study investigating the volatiles and color properties of basil leaves dried by microwave and freeze drying, eugenol was reported to have the largest peak in the freeze‐dried ones (Imaizumi et al., 2021). Similarly, the highest amount of eugenol was found in the FD sample, whereas the lowest amount was found in the microwave‐dried samples. According to Qu et al. (2019), drying duration significantly influenced the quantity of volatiles and the aroma of black tea. Tamaki et al. (2012) observed that microwave drying increased the porosity of the plant tissue. Imaizumia et al. (2021) reported that these effects may have influenced the retention power of volatiles in basil leaves. In the current study, we found that the amount of volatiles in microwave‐dried ones was generally lower than in purple basil leaves dried by freeze, sun, and convective.

Łyczko et al. (2020) researched the impact of various drying techniques (CD, vacuum–microwave drying, [VMD], and combined convective pre‐drying and vacuum–microwave final drying) on the EO compositions, volatiles, and organoleptic characteristics of Thai basil. They reported that, except for estragole, the percentage of all odor‐active components in Thai basil increased when different drying methods were applied; the largest increase was noted in basils convectively dried at 50°C and in ones where CD was started at 50°C and completed at 70°C. In contrast, methods consisting of or incorporating VMD had no discernible impact on the ratio of odor‐active compounds in the volatile profiles of the samples or reduced their role.

The primary key varies depending on temperature and time, leading to contrasting results; for instance, higher temperature increases volatile components but decreases processing time (Łyczko et al., 2020). Prusinowska and Śmigielski (2015) highlighted that enzymes that are secreted within cells are linked to chemoenzymatic reactions; these enzymes primarily catalyze hydrolysis or isomerization events; enzymes being released within the cells are primarily catalyzed. It is hard to forecast the exact result of a certain drying process due to the complexity of all these reactions; therefore, adequate experiments with suitable conditions and matrices should always be carried out.

### 3.6 Essential oil (EO) content in the purple basil leaves

The yield of EOs in basil leaf samples was obtained at the level of 0.20% ± 0.01%. The volatile compounds of the EOs isolated from the samples are presented in Table 3. The main constituents of the all samples were methyl cinnamate (11.68%–57.66%), linalool (0.02%–20.39%), 1,8‐cineole (0.12%–5.54%), α‐guanine (0.98%–15.84%), β‐elemene (2.20%–6.34%), γ‐cadinene (3.81%–22.75%), spatulenol (0.24%–7.76%), and γ‐eudesmol (1.52%–25.90%). Some of the main compounds of EOs isolated from the samples were comparable to our study investigating the EO components of purple basil leaves. The main EOs compounds of basil originating from many sites within Egypt (Assiut, Minia, and Beni Suef) were linalool, estragole, methyl cinnamate, bicyclosesquiphellandrene, eucalyptol, ‐bergamotene, eugenol, ‐cadinene, and germacrene D (Ahmed et al., 2019). Similarly, Chenni et al. (2016) reported linalool and methyl chavicol as the main constituents of EO extracted from Egypt. De Araújo Couto et al. (2019) reported linalool, methyl chavicol, neral, geranial, eugenol, and (E)‐methyl cinnamate as the major constituents of the EO of 24 basil genotypes. Ion et al. (2020) also showed that linalool, methyl chavicol, α‐epicadinol, and eugenol were the major compounds of the EO of fresh and freeze‐dried basil leaves. Gurkan and Hayaloglu (2023) reported that the main compounds of the EO obtained from fresh and shade‐dried organs (flowers, leaves, and stems) of purple basil were linalool, methyl cinnamate, methyl eugenol, eugenol, germacrene D, 1,8‐cineole, 𝛽‐elemene, 𝛼‐humulene, bicyclogermacrene, and 𝛼‐cadinol.

**TABLE 3:** Essential oil content in the Arapgir purple basil (*Ocimum basilicum* L.) leaves in fresh and dried at different conditions (%).

Name	RI^dbw^	RI^lit^	FB	FD	SD	CD45	CD50	CD55	MD350	MD460	MD600
(*E*)‐β‐Ionone‐5.6‐epoxide	668	–	N.D.	N.D.	N.D.	N.D.	N.D.	N.D.	1.29	0.24	N.D.
α‐Phellandrene	1025	–	N.D.	N.D.	N.D.	N.D.	N.D.	N.D.	0.48	N.D.	0.83
Hexanal	1080	1081^a^	N.D.	2.37	N.D.	N.D.	N.D.	N.D.	N.D.	N.D.	N.D.
Sabinene	1100	1111^h^	0.12	N.D.	0.46	N.D.	0.68	N.D.	N.D.	N.D.	N.D.
β‐Pinene	1102	1023^a^	N.D.	N.D.	0.80	N.D.	N.D.	N.D.	N.D.	N.D.	0.48
β‐Myrcene	1149	1143^d^	0.14	N.D.	0.27	N.D.	0.59	N.D.	N.D.	N.D.	N.D:
α‐Terpinene	1185	1167^c^	N.D.	N.D.	N.D.	N.D.	0.10	N.D.	N.D.	N.D.	0.42
dl‐Limonene	1189	1187^d^	0.20	N.D.	0.28	N.D.	0.50	N.D.	N.D.	N.D.	N.D.
1.8‐Cineol	1212	1209^a^	0.50	2.30	5.54	N.D.	4.41	N.D.	0.86	0.12	N.D.
(*E*)‐2‐Hexenal	1225	1209^f^	0.12	N.D.	N.D.	N.D.	N.D.	N.D.	N.D.	N.D.	N.D.
(*Z*)‐β‐Ocimene	1236	1238^e^	N.D.	N.D.	0.34	N.D.	0.38	N.D.	0.62	N.D.	1.04
γ‐Terpinene	1246	1240^a^	N.D.	N.D.	0.11	N.D.	N.D.	N.D.	0.28	N.D.	0.73
p‐Cymene	1272	1268^a^	N.D.	N.D.	N.D.	N.D.	N.D.	0.02	N.D.	0.04	N.D.
Terpinen‐4‐ol	1280	–	0.10	0.14	0.85	N.D.	0.91	N.D.	N.D.	0.12	N.D.
α‐Terpinolene	1283	1280^a^	N.D.	N.D.	N.D.	N.D.	0.16	N.D.	0.21	N.D.	N.D.
Octanal	1294	–	N.D.	N.D.	0.09	N.D.	0.17	N.D.	N.D.	N.D.	N.D.
3‐Hexen‐1‐ol	1393	1388^a^	N.D.	1.67	N.D.	N.D.	N.D.	N.D.	N.D.	N.D.	N.D.
Nerol	1433	–	0.34	N.D.	0.40	N.D.	0.52	N.D.	0.69	N.D.	1.17
1‐Octen‐3‐ol	1452	1456^h^	0.12	N.D.	0.30	N.D.	0.40	N.D.	N.D.	N.D.	N.D.
α‐Cubebene	1465	1463^a^	0.50	N.D.	1.58	N.D.	2.33	N.D.	3.46	N.D.	0.46
2.4‐Heptadienal. (*E.E*)	1476	1483^g^	N.D.	0.63	N.D.	N.D.	0.12	N.D.	N.D.	0.24	N.D.
(*E*)‐Sabinene hydrate	1477	1463^f^	0.89	N.D.	0.68	N.D.	0.50	N.D.	1.65	0.04	2.05
α‐Copaene	1504	1517^b^	0.34	N.D.	0.19	N.D.	0.36	N.D.	0.62	N.D.	0.65
β‐Bourbonone	1536	1541^i^	0.52	N.D.	0.50	N.D.		N.D.	0.65	N.D.	0.75
Camphor	1544	1538^a^	N.D.	N.D.	N.D.	N.D.	0.83	N.D.	0.84	N.D.	N.D.
Linalool	1549	1555^a^	13.58	N.D.	20.39	11.50	18.12	0.02	8.63	4.69	7.58
Linalool‐l	1550	–	N.D.	30.78	N.D.	N.D.	N.D.	N.D.	N.D.	N.D.	N.D.
(*E*)‐α‐Bergamotene	1594	1595^h^	2.09	N.D.	0.53	N.D.	N.D.	0.02	3.23	0.08	4.72
β‐Elemene	1601	1587^b^	6.12	N.D.	2.84	N.D.	3.30	2.20	4.43		6.34
α‐Guaiene	1607	1588^c^	1.89	N.D.	0.98	N.D.	2.55	3.81	2.19	15.84	2.46
(*E*)‐β‐Caryophyllene	1619	1649^i^	N.D.	N.D.	0.53	N.D.	0.59	N.D.	N.D.	N.D.	N.D.
Caryophyllene	1622	1608^g^	0.99	N.D.	N.D.	N.D.	N.D.	17.39	0.68	N.D.	1.27
Aromadendrene	1626	1643^c^	N.D.	N.D.	N.D.	N.D.	0.14	N.D.	N.D.	N.D.	N.D.
β‐Cedrene	1627	–	N.D.	N.D.	0.54	N.D.	N.D.	N.D.	0.28	N.D.	0.40
(*E*)‐β‐Farnesene	1669	1671^h^	1.57	N.D.	1.75	N.D.	2.46	N.D.	0.82	N.D.	1.11
p‐Menth‐1‐en‐8‐ol	1688	–	N.D.	2.44	N.D.	N.D.	0.09	N.D.	N.D.	0.08	N.D.
α‐Humulene	1694	1681^h^	3.99	N.D.	2.47	N.D.	N.D.	N.D.	2.59	N.D.	5.05
Germacrene	1733	1724^e^	4.21	N.D.	N.D.	N.D.	N.D.	N.D.	N.D.	16.76	0.69
α‐Bulnesene	1739	–	N.D.	N.D.	N.D.	N.D.	N.D.	8.18	N.D.	N.D.	N.D.
β‐Selinene	1752	1707^i^	N.D.	N.D.	N.D.	N.D.	0.13	N.D.	2.02		1.34
Bicyclogermacrene	1761	–	2.50	N.D.	N.D.	N.D.	N.D.	N.D.	0.84	11.11	0.86
γ‐Cadinene	1784	1749^e^	5.88	N.D.	3.89	N.D.	22.75	N.D.	12.10	3.81	5.80
Nerolidol	1808	–	1.13		0.97	N.D.	1.33	N.D.	1.09	0.04	
Geraniol	1853	1837^a^	0.62	0.35	0.73	N.D.	0.99	N.D.	1.86	0.68	2.57
(*Z*)‐Calamenene	1862	–	0.38	N.D.	0.40	N.D.	N.D.	N.D.	0.80	N.D.	1.23
Benzyl alcohol	1894	1870^f^	N.D.	N.D.	N.D.	N.D.	N.D.	N.D.	N.D.	0.08	N.D.
Phenylethyl alcohol	1934	1906^i^	N.D.	0.14	N.D.	N.D.	N.D.	N.D.	N.D.	0.04	N.D.
β‐Ionone	1967	1912^a^	N.D.	N.D.	N.D.	N.D.	0.09	N.D.	N.D.	0.12	N.D.
Methyleugenol	2017	2011^h^	0.18	0.63	N.D.	N.D.	3.47	N.D.	1.94	0.52	1.63
Hotrienol	2023	–	N.D.	N.D.	N.D.	N.D.	N.D.	N.D.	0.86	N.D.	N.D.
β‐Cyclocitral	2029	–	N.D.	N.D.	0.41	N.D.	N.D.	N.D.	N.D.	N.D.	N.D.
Caryophyllene oxide	2031	–	0.66	N.D.	0.09	N.D.	N.D.	N.D.	N.D.	N.D.	N.D.
α‐Humulene oxide	2054	2038^f^	0.46	N.D.	N.D.	N.D.	N.D.	N.D.	1.11	N.D.	N.D.
β‐Sesquiphellandrene	2077	2035^j^	0.89	N.D.	N.D.	N.D.	1.50	8.69	0.44	N.D.	0.61
α‐Curcumene	2078	–	N.D.	N.D.	N.D.	N.D.	N.D.	N.D.	0.26	N.D.	0.36
2‐Propenoic acid 3‐phenyl‐ methyl ester	2104	–	43.21	57.66	32.69	54.70	11.68	54.38	25.16	18.68	29.10
Spathulenol	2178	2120^f^	2.09	N.D.	4.92	N.D.	7.13	N.D.	6.44	0.24	7.76
Eugenol	2179	2172^h^	N.D.	N.D.	3.06	N.D.	3.10	5.25	3.12	N.D.	N.D.
α‐epi‐Muurolol	2188	–	N.D.	N.D.	4.63	N.D.	4.97	N.D.	N.D.	7.54	N.D.
α ‐Eudesmol	2215	2220^f^	N.D.	N.D.	1.52	25.90	1.68	N.D.	2.09	N.D.	3.17
Isophytol	2279	–	0.54	N.D.	0.37	N.D.	0.25	N.D.	0.96	N.D.	1.69
Longifolenaldehit	2425	–	N.D.	N.D.	1.10	N.D.	N.D.	N.D.	1.56	18.68	N.D.
Ethyl linoleolate	2557	–	3.06	N.D.	1.02	N.D.	0.14	N.D.	2.06	N.D.	3.59

*Note*: FB, fresh sample; FD, freeze‐dried sample; SD, sun‐dried sample; CD45, convection‐dried sample at 45°C; CD50, convection‐dried sample at 50°C; CD55, sample at 55°C; MD350, microwave‐dried sample at 350 W; MD460, microwave‐dried sample at 460 W; MD600, microwave‐dried sample at 600 W; RI^dbw^, retention indices calculated on DB‐WAX column using alkane standards; RI^lit^, retention indices according to literature.

^a^ Sonmezdag et al. (2018).

^b^Usami et al. (2013).

^c^Roitman et al. (2011).

^d^Gurkan and Hayaloglu (2017).

^e^Jordan et al. (2017).

^f^Lee et al. (2005).

^g^Mahmoud et al. (2022).

^h^Kang et al. (2012).

^i^Sevindik et al. (2022).

^j^Gurkan and Hayaloglu (2023).

The highest levels of methyl cinnamate (57.66%) and linalool‐l (30.78%) were found in FD. Linalool was found more frequently (20.39%) in SD compared to the other samples. For CD50, γ‐cadinene (22.75%) was found to be in high content. The highest germacrene value (16.76%) was observed in MD460.

**FIGURE 3:**
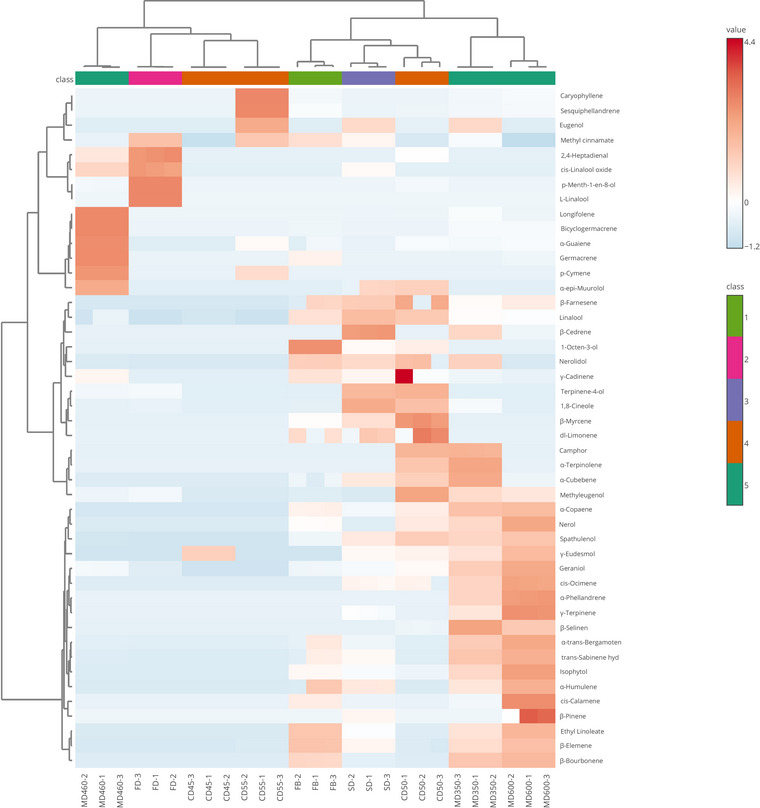
The heat map of volatiles in the essential oil of samples. Rows: Volatile organic compounds identified; columns: basil leaves dried by different methods and FB. The color scale, ranging from low abundance (blue) to high abundance (red), may be inferred from the relative abundance in the display of heat maps (basil leaf codes are as follows: FB, fresh sample; FD, freeze‐dried sample; SD, sun‐dried sample; CD45, convection‐dried sample at 45°C; CD50, convection‐dried sample at 50°C; CD55, convection‐dried sample at 55°C; MD350, microwave‐dried sample at 350 W; MD460, microwave‐dried sample at 460 W; MD600, microwave‐dried sample at 600 W).

The samples were divided into two subclusters by heat mapping. The data of the EOs volatiles isolated from purple basil leaves are shown in Figure 3. MD460 in the first cluster and FB in the second cluster are different from the other samples. Other dried purple basil leaves are intrinsically similar. The content of longifolene, bicyclogermacrene, α‐guaiene, and germacrene was highest in MD460. The contents of 2,4‐heptadienal, p‐menth‐1‐en‐8‐ol, and l‐linalool were highest in FD. The content of caryophyllene and sesquiphellandrene was highest in CD55. γ‐Cadinene was found in the highest concentrations in CD50. The concentration of β‐pinene was highest in MD600.

*O. basilicum* species grow in many parts of the world and differ in their morphological structure, so that the content and chemical composition of the EO vary. Stanojevic et al. (2019) stated that linalool (31.6%), methyl chavicol (23.8%), β‐elemene (6.9%), and α‐bulnesene (4.5%) were the most important compounds in basil samples from the northwest of the Republic of Srpska, Bosnia, and Herzegovina. One of the basil samples from Malatya with two different genotypes contained high levels of linalool (37.7%–60.2%), 1,8‐cineole (0.2%–14.5%), eugenol (3.1%–21.1%), and δ‐cadinene (7.4–8. 7.4%–8.7%), whereas the other has high levels of citral (56.6%–65.6%), linalool (3.2%–5.3%), α‐bisabolene (2.1%–3.4%), geraniol (1.0%–3.9%), and methyl eugenol (0.8%–3.3%) (Telci et al., 2006). In addition, basil from Antalya and Anamur regions was reported to contain a high amount of EO of methyl cinnamate (58.6%–63.1%) (Gunay & Telci, 2017). Although the EO ratios of methyl eugenol (0.18%–3.47%), geraniol (0.35%–2.57%), and methyl cinnamate (11.68%–57.66%) in the purple basil leaves from Arapgir used in our study were similar to those in the aforementioned study, the EO ratios of linalool (0.02%–20.39%) and 1,8‐cineole (0.12%–5.54%) were lower compared to this study. It is hypothesized that this difference could be due to seasonal differences and different harvest times.

In a study investigating the effects of different drying methods (sun, shade, and oven at 40 and 60°C, microwave drying at 500 W, freeze drying) on the EO yield and composition of purple and green *O. basilicum* grown in Iran, the main components of the EO of the purple varieties were methyl chavicol (65.63%), linalool (6.11%), and *trans*‐α‐bergamotene (4.09%), whereas the major compounds of the EO of the green varieties were geraniol (17.61%) and neral (15.04%) (Pirbalouti et al., 2013). It was also found that many monoterpene hydrocarbons disappeared with increasing oven temperature and microwave power. As drying temperature increased, oxygenated sesquiterpenes (epi‐α‐cadinol), sesquiterpene hydrocarbons (β‐bisabolene and β‐elemene), and oxygenated monoterpenes (geranyl acetate, α‐terpinyl acetate, thymol, and α‐terpineol), which were absent in fresh and green varieties, increased. Similar to the aforementioned study, monoterpene hydrocarbons were observed to disappear in FD, CD45, CD55, and MD460 after drying; in contrast to the same study, monoterpene hydrocarbons increased in samples SD, CD50, MD350, and MD600. Moreover, similar to the same study, oxygenated sesquiterpenes were observed to increase in the leaves due to the sun, convection, and microwave drying methods compared to FB. Pirbalouti et al. (2013) reported that drying increases the amount of water removed from the tissue surface; some EO components may increase slightly, and some monoterpenes and phenylpropanoids are converted to sesquiterpenes with increasing temperature. They also found that OD at 40°C and freeze drying are the most suitable methods for EO yield. In contrast to the findings of the mentioned research, the concentration of *M. haplocalyx* leaves EO decreased significantly at the different temperatures of hot air drying (35, 45, and 55°C) compared to the fresh leaves (Guo et al., 2022). Another study on the drying of *Thymus daenensis* leaves showed that as the drying temperature increased, the amount of EO reduced (Mashkani et al., 2018).

## 4 CONCLUSION

In this study, purple basil leaves were dried using different methods, including freeze drying, sun‐drying, convective drying, and microwave drying. The results showed that the drying methods significantly affected the color, chemical composition, and volatiles. Freeze drying was the most effective method to preserve anthocyanins and volatile compounds, whereas microwave drying minimized the loss of TPC. A total of 73 volatile compounds and 65 EOs were identified, with linalool, 1,8‐cineole, and methyl cinnamate being the most important compounds. These three compounds were also significantly affected by the drying methods. The results will contribute to the selection of the most suitable drying methods, which are crucial for the production and subsequent commercialization of geographically registered Arapgir purple basil leaves.

## AUTHOR CONTRIBUTIONS

**Kadriye Altay**: Conceptualization; methodology; software; data curation; investigation; formal analysis; writing—original draft. **Safiye Nur Dirim**: Conceptualization; supervision. **Ali Adnan Hayaloglu**: Conceptualization; methodology; software; data curation; investigation; validation; formal analysis; supervision; funding acquisition; visualization; project administration; writing—review and editing.

## CONFLICT OF INTEREST STATEMENT

The authors declare no conflicts of interest.

## Data Availability

The data are available from the corresponding author upon suitable request.
